# Long-Term Urban Market Dynamics Reveal Increased Bushmeat Carcass Volume despite Economic Growth and Proactive Environmental Legislation on Bioko Island, Equatorial Guinea

**DOI:** 10.1371/journal.pone.0134464

**Published:** 2015-07-31

**Authors:** Drew T. Cronin, Stephen Woloszynek, Wayne A. Morra, Shaya Honarvar, Joshua M. Linder, Mary Katherine Gonder, Michael P. O’Connor, Gail W. Hearn

**Affiliations:** 1 Department of Biology, Drexel University, Philadelphia, Pennsylvania, United States of America; 2 Department of Biodiversity, Earth and Environmental Science, Drexel University, Philadelphia, Pennsylvania, United States of America; 3 Bioko Biodiversity Protection Program, Malabo, Bioko Norte, Equatorial Guinea; 4 Department of Electrical and Computer Engineering, Drexel University, Philadelphia, Pennsylvania, United States of America; 5 School of Global Business, Arcadia University, Glenside, Pennsylvania, United States of America; 6 Department of Biology, Indiana University - Purdue University Fort Wayne, Fort Wayne, Indiana, United States of America; 7 Department of Sociology and Anthropology, James Madison University, Harrisonburg, Virginia, United States of America; Institute of Agronomy, University of Lisbon, PORTUGAL

## Abstract

Bushmeat hunting is extensive in west and central Africa as both a means for subsistence and for commercial gain. Commercial hunting represents one of the primary threats to wildlife in the region, and confounding factors have made it challenging to examine how external factors influence the commercial bushmeat trade. Bioko Island, Equatorial Guinea is a small island with large tracts of intact forest that support sizeable populations of commercially valuable vertebrates, especially endemic primates. The island also has a low human population and has experienced dramatic economic growth and rapid development since the mid-1990’s. From October 1997 – September 2010, we monitored the largest bushmeat market on Bioko in Malabo, recording over 197,000 carcasses for sale. We used these data to analyze the dynamics of the market in relation to political events, environmental legislation, and rapid economic growth. Our findings suggest that bushmeat hunting and availability increased in parallel with the growth of Equatorial Guinea’s GDP and disposable income of its citizens. During this 13-year study, the predominant mode of capture shifted from trapping to shotguns. Consequently, carcass volume and rates of taxa typically captured with shotguns increased significantly, most notably including intensified hunting of Bioko's unique and endangered monkey fauna. Attempts to limit bushmeat sales, including a 2007 ban on primate hunting and trade, were only transiently effective. The hunting ban was not enforced, and was quickly followed by a marked increase in bushmeat hunting compared to hunting rates prior to the ban. Our results emphasize the negative impact that rapid development and unenforced legislation have had on Bioko’s wildlife, and demonstrate the need for strong governmental support if conservation strategies are to be successful at preventing extinctions of tropical wildlife.

## Introduction

The harvesting of bushmeat for human consumption is common throughout the developing world as a means for people to meet many of their dietary and livelihood needs [1–3], and subsistence bushmeat hunting is particularly prevalent in western and central Africa [4, 5]. In many parts of the African moist forest zone, however, bushmeat hunting has evolved from a subsistence practice to an unsustainable, commercialized business [6–8] driven by increasing access to firearms, a lack of alternative protein sources, widespread industrial logging leading to increased infrastructure development and greater access to remote forests, and ultimately, a quickly growing urban human population that fuels increased demand [9–11]. In the relatively densely populated Gulf of Guinea forests of central Africa, demand for bushmeat is particularly high and hunting is highly commercialized [1, 8, 12, 13]. As a result, bushmeat hunting is considered among the most significant threats to the conservation of biological diversity in the tropics [8–10, 14, 15], particularly in western and central Africa where increased hunting pressure resulting from commercialization has contributed to the local extirpation of many rainforest mammal species [10, 15–20]. Widespread bushmeat hunting also represents a serious threat to human populations. The transmission of zoonotic pathogens via human contact with infected bushmeat [21, 22], as well as the decline and/or loss of a cheap and readily available protein source [23, 24], both represent major public health concerns with long-term ramifications.

Despite the serious threats the bushmeat trade represents to wildlife and to humans, our understanding of the factors that govern the bushmeat trade remains incomplete and disjointed, and as a result, solutions to mitigate the crisis have been applied with very limited success. Poverty alleviation, for example, has been suggested as a solution to decrease the supply of and demand for bushmeat, with rising incomes leading to a decline in reliance upon environmental resources for survival; however, development objectives focused on poverty alleviation alone have not reduced consumption [25–27], and many studies have indicated a consistent link between increased wealth and greater consumption of wildlife [28–30]. The results are not uniformly negative though; for example, national economic development has led to a decrease in the intensity and extent of hunting in a rural area in mainland Equatorial Guinea [31]. Another common approach has been blanket criminalization, but this too has often been unsuccessful in reducing consumption [32–36]. A better understanding of the dynamic response of the bushmeat economy to economic development and government legislation efforts is critical for targeting more effective interventions.

The bushmeat market in Malabo, the capital and commercial center of Equatorial Guinea, on Bioko Island, provides a unique opportunity for studying the bushmeat trade in central Africa. Bioko contains an insular subset of Gulf of Guinea fauna, including seven species of threatened monkeys [37, 38], bushmeat hunting is extensive [29, 38–40], and the market supply chain [producer (hunter)–intermediary (taxi-driver)–market vendor] is similar to that of other regional markets [39, 41]. However, the Malabo market differs from others in that it lacks many of the confounding factors that constrain inferences derived from market-based studies. The market operates in a small, contained system, supplied by limited and easily enumerated transit routes [41], with the majority of consumption restricted to Malabo [39]. Hunting is largely conducted by migrant commercial hunters from Rio Muni, the mainland sector of Equatorial Guinea, almost exclusively for profit [39, 42–44], and although there has been much recent urban development and sprawl surrounding Malabo, other anthropogenic factors (e.g., deforestation) have had limited impact on much of the island’s forests due to difficult terrain [45]. Furthermore, alternative protein sources are readily available throughout the towns and villages of Bioko, including fish, pork, chicken, and beef, and numerous studies have corroborated that bushmeat is neither a significant contributor of protein [29, 43, 44, 46], nor can it possibly fulfill more than a fraction of the dietary and economic needs of the general population [43, 46]. The population of Malabo is not dependent on bushmeat [46], and consumption may be associated with wealth and status [43], especially in the case of primates [46]. Reid et al. [43] reported that the median income for bushmeat consumers was 3–4 times the per capita GDP in 2001. Conversely, Albrechtsen et al. [46] found that income was negatively correlated with household bushmeat consumption in Malabo, and on the outskirts of Malabo, Grande Vega et al. [44] reported that consumption of bushmeat protein was not affected by income. Malabo consumers do exhibit a preference for fresh meat, with a slight bias towards bushmeat [43], but fish is also an important protein source [44]. The majority of bushmeat consumers are of the Fang ethnic group, originally from Rio Muni, but the indigenous Bubi group also make up a quarter of the market [43], and there is no difference between the amount of bushmeat protein consumed between the two ethnicities [44]. Government attempts to regulate the bushmeat trade in Equatorial Guinea have focused on regulating hunter behavior by banning hunting within protected areas [47] and prohibiting take of specific threatened taxa (e.g. monkeys) [48]. Still, illegal hunting occurs extensively throughout federally protected areas [44, 49], as Equatorial Guinea has yet to implement any management strategy or enforcement regime, resulting in effectively open access forests.

In this study, we used long-term data on animal carcass numbers for sale in the Malabo bushmeat market gathered by the Bioko Biodiversity Protection Program (BBPP), in collaboration with the National University of Equatorial Guinea (UNGE). The unique nature of the Malabo market and the scope of the dataset allowed us to investigate how market dynamics (i.e., numbers and species composition of animals for sale in the market) have changed over time. We examine the relation between rapid economic development and carcass rates, specifically between income and overall carcass volume, as well as how carcass volume has responded to externalities, such as political events and environmental legislation, and the efficacy of a trade ban on primates enacted on the Malabo bushmeat market in late 2007 [50]. Our results highlight the immediate need for effective conservation measures on Bioko Island and the importance of government support and long-term planning for environmental and conservation legislation.

## Methods

### Study area

**Fig 1:**
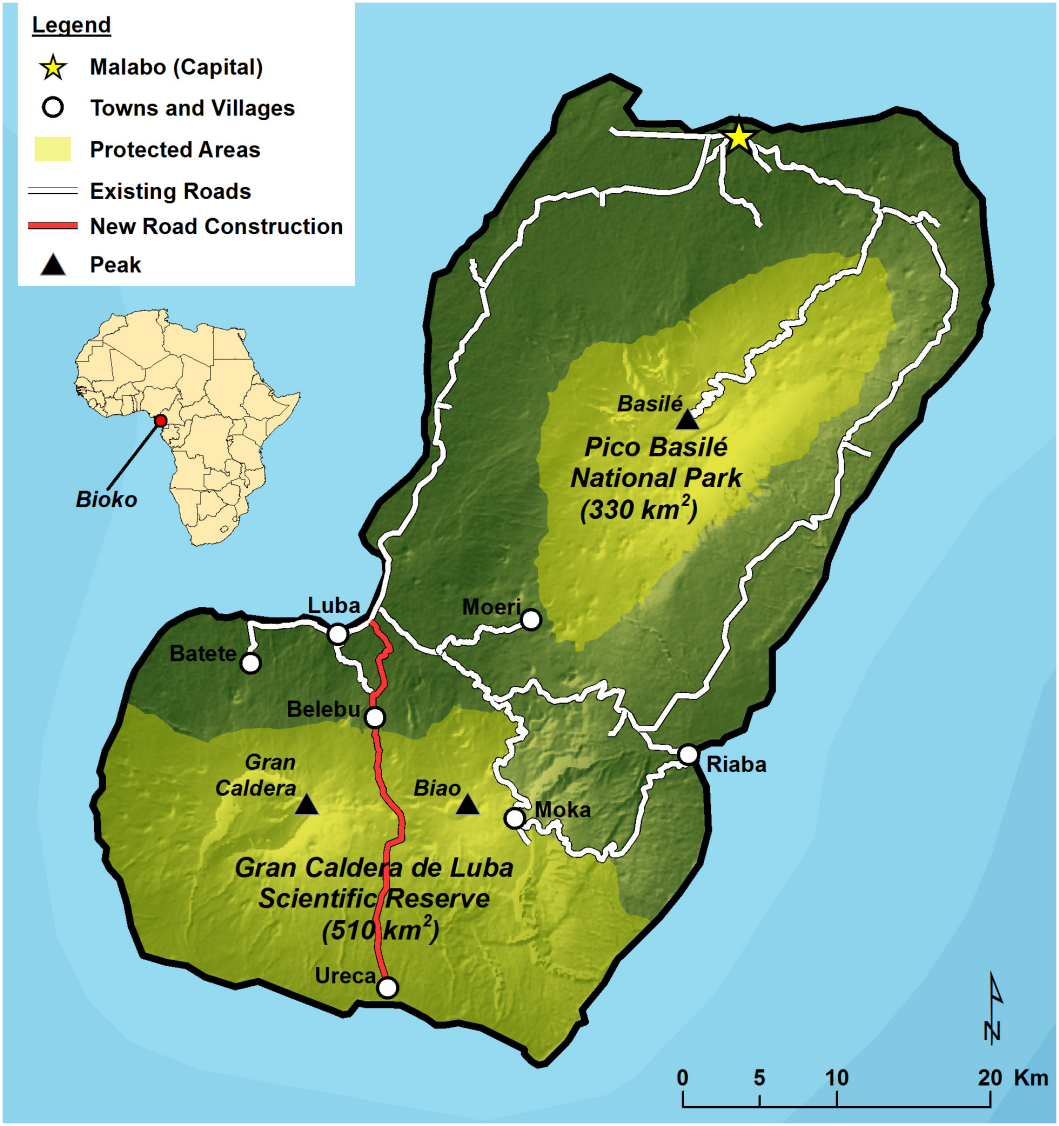
Distribution of major human settlements, main roads, and protected areas on Bioko Island. Also shown is Ureca, the only permanent human settlement entirely within a protected area on Bioko.

Bioko Island, Equatorial Guinea (2,017 km^2^) is a volcanic, continental island in the Gulf of Guinea island chain located 37 km off the coast of Cameroon (Fig 1). The steeply vertical island rises to three volcanic peaks (Pico Basilé [3,011 m] in the north; the Gran Caldera de Luba [2,261 m] in the southwest; and Pico Biao [2,009 m] in the southeast) all within 15 km of the coast. The climate is tropical equatorial with distinct dry (Nov-Mar) and wet (Apr-Oct) periods combined with high variation in localized annual rainfall amounts, ranging from over 10 m in the south to 2 m in the north [51]. Bioko forms part of the West African Forests biodiversity hotspot [52], and encompasses two ecoregions, the Cross-Sanaga-Bioko coastal forests and, at higher elevations, the Mount Cameroon/Bioko montane forests [53]. The human population is concentrated at low elevations and in the northern sector, with population densities highest in Malabo (>100 inhabitants km^2^) [46]. The population is estimated to have more than doubled since the mid-1990’s following the discovery of offshore oil, which led Equatorial Guinea through a long period of rapid economic growth [54, 55]. However, much of Bioko still supports no permanent human settlement, with few villages scattered throughout the areas bordering Bioko’s two protected areas, Pico Basilé National Park (PBNP) (330 km^2^) in the north and the Gran Caldera de Luba Scientific Reserve (GCSR) (510 km^2^) comprising the southern 30% of the island. Ureca (< 80 individuals), in the GCSR, is the only village located entirely within a protected area. Recent construction of a highway that bisects the GCSR and connects the village of Ureca with Bioko’s second largest city, Luba, now allows vehicle access for the first time to the previously isolated southern extent of Bioko.

### Ethics Statement

Permission for this study was granted by the government of Equatorial Guinea. All research activities were conducted in collaboration with the National University of Equatorial Guinea.

### Market Census

Under the auspices of UNGE, two local contractors recorded data detailing Malabo bushmeat market activity from October 1997 through September 2010. The market operated year-round, Monday-Saturday, with occasional Sunday activity until approximately May 2010, after which the market sporadically closed on Tuesdays for cleaning. Data were recorded as game arrived from 07:00 to 12:30 and included age (adult, juvenile), condition (alive, smoked, fresh), capture method, sex, taxon, origin (island, import), and price in Central African Francs (FCFA; approximately 500 FCFA to 1 USD). Data collectors recorded both the carcass’s binomial name and Spanish common name, and classified carcasses into five predefined taxonomic groups: avian, primate, reptile, rodent, and ‘other mammals’ (non-primate, non-rodent), from which we further separated ungulates for analyses. For capture method, data collectors determined if the animal was trapped or killed using a shotgun. Carcass origin information included whether the carcass was imported—from Rio Muni or Cameroon—or captured from Bioko Island and, when possible, the region of capture, determined by the individual intermediary bringing the carcasses to market or the vendor selling the specific carcasses. The majority of meat in the Malabo market is sold on the day it is taken to market [41], thus it is unlikely that more than a small fraction of carcasses may have been missed or counted twice despite data collectors’ best efforts (e.g. if a carcass was not sold and held overnight for sale on the subsequent day).

### Data Preparation

Carcass data were categorized by taxon, capture method, and geographic origin (mainland or Bioko). Species enumerated less than 75 times over the course of the study (e.g., *Pan troglodytes*, sporadically imported from the mainland) were omitted due to rarity, which may also have led to incorrect identification of the carcasses due to our data collectors’ unfamiliarity with the particular species. “Carcass rates” were generated for each species and aggregation by dividing the total number of carcasses recorded per month by the total number of days that month the market was surveyed. To account for data loss due to a fire in February 2001, data for January and March 2001 were averaged. Visual inspection of the total island and primate data sets suggested three distinct periods of market behavior. We hypothesized that three known major government conservation/management interventions may have driven the changes in market behavior—i.e., changes in frequency, trends, and seasonality—during these periods: (1) March 2002—a Biodiversity Roundtable sponsored by Conservation International (CI), BBPP, and UNGE; (2) November 2003- the passing of law number 7/2003 prohibiting hunting within protected areas; and (3) October 2007-the passing of decree number 72/2007 prohibiting the hunting, sale, or consumption of primates. To confirm these interventions marked significant shifts in market behavior, an intervention model was implemented [56]. All subsequently described analyses were performed using only data from carcasses originating from Bioko in order to control for catchment size and to limit the impact of confounding factors that arise from the use of market data [57].

### Intervention Model

A simple means to compare time series behavior before and after a particular intervention may involve breaking the series into two groups and implementing a t-test for group means; however, this would require violation of two assumptions: (1) the data consist of independent observations, and (2) the data were homoscedastic. For our data to be appropriately analyzed, we needed to account for autocorrelation and the possibility that variance changed significantly pre- or post-intervention; hence, we implemented an intervention model, a model often found in econometrics or environmental impact analysis, but not conservation. Intervention model fitting and parameter evaluation adhered to Cryer and Chan [58]. We designated notable events as external market interventions that potentially interrupted the normal behavior of the time series. Identifying changes in market dynamics allowed us to establish distinctive periods within the series.

Because hunting had a large effect on primates, which as charismatic and threatened fauna were the major drivers of conservation efforts, the primate series was used in the intervention model. Before interventions could be evaluated, an unperturbed portion of the series was modeled (the Noise model) in order to infer changes caused by interventions. Square root transformed primate carcass rates prior to the primate hunting ban (before 11/2007) were fit with a seasonal autoregressive integrated moving average (SARIMA) model [59]. We hypothesized that the ban had the most significant effect on the behavior of the series, and hence, was chosen as the breakpoint for pre-intervention data, despite four additional interventions spanning the series prior to the passing of the ban. Model parameters were chosen by evaluating residual normality, autocorrelation at various lags, and a Ljung-Box test statistic.

Five interventions were analyzed as external influences with the same pre-intervention SARIMA model, but now fitted for the full series (through September 2010) using the function ‘arimax’ from the R package ‘TSA’ [60]. The five interventions were (1) a January 1998 failed coup attempt (manifested in February); (2) the passage of law number 4/2000 in May 2000, updating the designation of protected areas [61]; (3) the CI/BBPP/UNGE Bushmeat Roundtable in March 2002; (4) the passage of law number 7/2003, an environmental regulatory law, in November 2003 [62]; and (5) decree number 72/2007 in October 2007 (manifested in November) banning the hunting of primates [50] (Table 1). Model fitting indicated the May 2000 protected areas law did not significantly affect the carcass rate; hence, it was removed. The intervention functions are listed in Table 1.

**Table 1:** Parameter estimation for intervention functions.

	Intervention				
Intervention Type (*notation*)	2/1998	5/2000	3/2002	11/2003	11/2007
**Step** *wS*_*t*_	---	---	0.457 (0.212)	-1.001 (0.308)	3.037 (0.636)
**Pulse** *wP*_*t*_	-1.357(0.295)	---	-0.625 (0.308)	3.037 (0.636)	-5.202 (0.654)
**Time Lag $\frac{1}{\left(1-wB\right)}$**	---	---	---	---	0.945 (0.013)

*B* is a backshift operator such that ***BP***_***t***_ = ***P***_***t-1***_; ***w*** are weights fitted by the model for a given function and are reported as estimate (standard error). ***P***_***t***_ is a pulse function, a momentary deviation that rapidly returns to baseline. ***S***_***t***_ is a step function, a permanent shift in the mean of the series. Time lag determines the number of time steps required for the altered mean, caused by the pulse function, to return to baseline.

### Trend Analysis

The data were first broken into periods reflecting the change in market behavior confirmed by the intervention analysis. The January 1998 coup attempt was excluded from consideration as a period breakpoint since its effect was short-lived. Although both the March 2002 bushmeat roundtable and the November 2003 environmental legislation significantly affected primate carcass rates, we selected a breakpoint of April 2003 for the Early/Pre-ban break point due to the behavior of the overall market. We believed this breakpoint to be more reflective of the overall market behavior, and not only that of the primate group. The resulting periods were: 1) October 1997 through March 2003 (“Early”), 2) April 2003 through October 2007 (“Pre-ban”), and 3) November 2007 through September 2010 (“Post-ban”).

Carcass rate records were then partitioned into seasonal and temporal components using the following additive model:

$$Y_{t}=T_{t}+S_{t}+E_{t}$$ (1)

where

*t* = month number from October 1997—September 2010

*Y*_*t*_ = carcass count per market day

*T*_*t*_ = the long-term trend component

*S*_*t*_ = the seasonal component

*E*_*t*_ = monthly (error) variation component.

Each record was first smoothed with first order locally weighted regression (loess) with 24 monthly points in the smoothing coefficient to isolate long-term trends [63]. Residuals were then smoothed with second order loess over 6 monthly points to capture the seasonal and error components. Parallel analyses using different time series techniques, including the highly constrained seasonal-trend decomposition (STL) [64] and X-12 ARIMA algorithm [65] gave similar results.

We performed time trend analyses on deseasonalized monthly average carcass rates (T_t_ + E_t_) for each taxon and each period. Because seasonal variation in carcass rates could obscure long-term trends, and was the subject of a separate analysis [66], the seasonal component was removed for each taxon. Quadratic regression of carcass rates (carcass market day^−1^) on serially numbered months was used to permit curvature of trends within periods. For each taxon and period, we also fit a reduced linear regression, and quadratic terms were retained only when the linear model was significantly less predictive than the quadratic model [67]. Average change in carcass rate per month (“slope[s]”) and 95% confidence intervals were calculated for each taxon and period. Regressions without significant quadratic terms were the slope of the line relating carcass rate to month. When the quadratic term was significant, the slope at the midpoint of the period and its confidence interval were calculated using linear model theory [68]. Note the distinction from hereafter between references to “rates”, which denote carcasses per market day averaged across each month, and “slopes”, which exclusively refer to the regression of carcass rates across periods, and hence are a measure of the rate of change of the aforementioned rates.

Slopes [change in carcass rate per month (carcass market day^−1^ month^−1^)] for different taxa were almost proportional to the fractional representation of the taxon in the total market carcass rate and were normalized for this effect to be directly compared. Normalization can be performed either by dividing each slope by the fraction of the total market carcass rate comprised by the focal taxon or by dividing the slope by the ratio of the standard deviations for the entire time series of the respective taxa and total carcasses [69]; both methods gave similar results. Here we present slopes normalized by the ratio of standard deviations because it is slightly more conservative when used on uncommon species.

We performed a two-factor analysis of variance (ANOVA) to determine how capture method and period affected capture rate. To reduce residual heteroscedasticity, we log transformed capture rates, then accounted for remaining heteroscedasticity by subjecting models to a generalized least squares (GLS) linear regression model that allowed a different variance structure for each level within each respective factor [70]. Lastly, correlation was calculated between rates of trapped and shotgunned carcasses, with 95% confidence intervals generated via a bootstrap procedure.

In markets where bushmeat is considered a luxury good, consumption has been positively correlated with income [30]. To test whether bushmeat is a luxury good on Bioko, we compared denoised monthly average carcass rates (T_t_ + S_t_) with income via linear regression. Due to a lack of reliable income data from Equatorial Guinea, we used the monthly European Brent Spot Price for crude petroleum in USD per barrel [71] as a proxy for income following Morra et al. [29], given that approximately 90% of Equatoguinean gross domestic product is derived from hydrocarbons [72]. Oil prices were then regressed against total, shotgunned, and trapped carcasses in each period to determine correlation.

We used *R* (v2.14.2; R Core Development Team 2007) [73] and MATLAB (R2011a) [74] to conduct all statistical analyses and produce all figures.

## Results

### General market description

**Fig 2:**
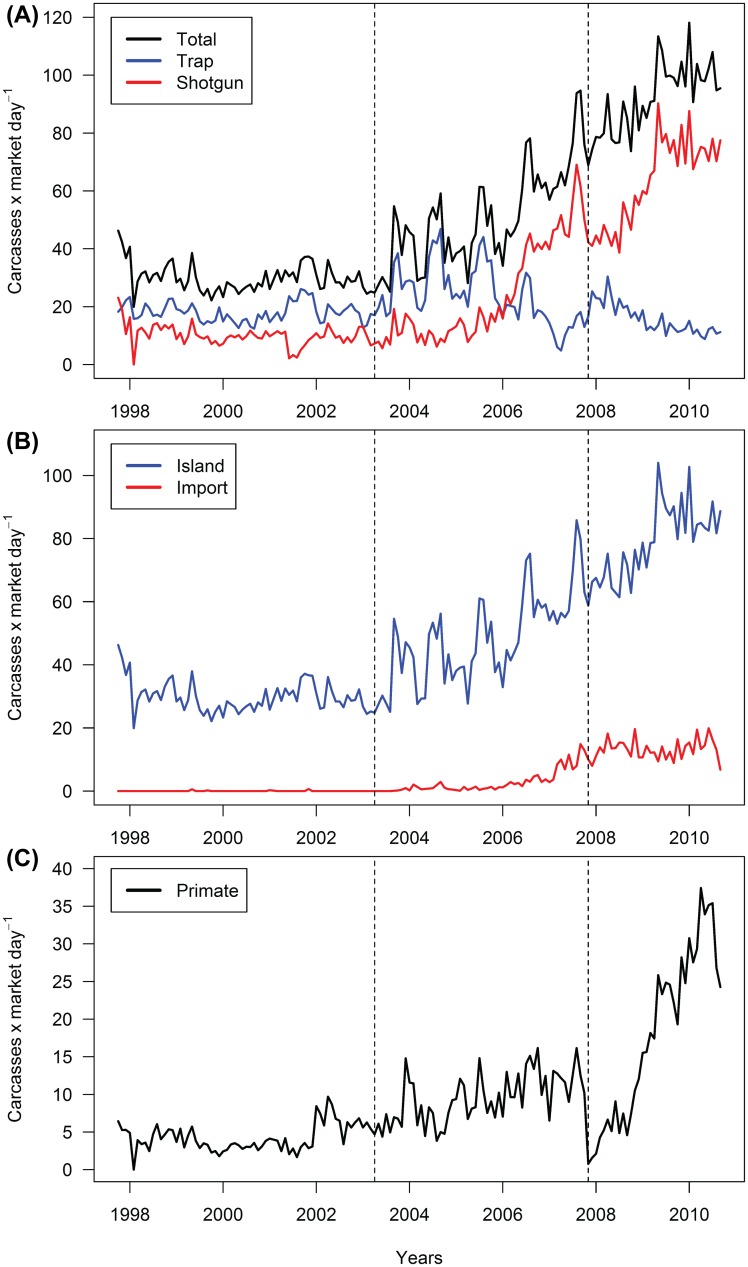
Time series plots of the carcass rates (carcasses/market day). Shown are the (A) total number of carcasses and the number of carcasses captured by shotgun or trap, (B) the number of domestic (island), and (C) the number of primate carcasses. Vertical lines delineate periods. The transition between Pre-ban and Post-ban also coincides with the passing of the October 2007 primate hunting ban.

We enumerated 196,892 carcasses over 3,758 market days in the Malabo market between October 1997 and September 2010, and identified 28 unique species: 3 avian, 8 primate, 4 reptile, 5 rodent, 3 ungulates, and 5 ‘other’ mammals (carnivores, hyrax, and pangolins) after omissions of rare taxa (enumerated less than 75 times) (Table 2). Most carcasses (81.9%) were fresh (recently killed) when they arrived in the market and only 12.6% were smoked. Few animals (5.1%) reached the market alive. Market growth was consistent over time, as the mean number of carcasses per market day increased progressively over the three periods: 29.94 (SD 4.94), 50.46 (SD 16.65), and 91.86 (SD 12.11) carcasses/day, respectively (Fig 2).

**Table 2:** Summary of the species, total carcass number and biomass (kg) for carcasses entering the Malabo market from Oct. 1997—Sept. 2010.

Species Grouping	Species	Common Name	Status^†^	Mass (kg)	Source^‡^	n	Biomass (kg)
Artiodactyla							
	*Cephalophus ogilbyi ogilbyi* ^a^	Ogilby's Duiker	LC	19.50	1.00	9,381	182,929.50
	*Philantomba monticola* ^a^	Blue Duiker	LC	4.90	1.00	49,318	241,658.20
	*Potamochoerus porcus* ^d^	Red River Hog	LC	80.00	0.00	266	21,280.00
	Group Totals					58,965	445,867.70
Aves							
	*Ceratogymna atrata* ^b^	Black-casqued Hornbill	LC	1.50	1.00	1,473	2,209.50
	*Corythaeola cristata* ^b^	Great Blue Turaco	LC	1.20	1.00	2,259	2,710.80
	*Gypohierax angolensis* ^c^	Palm-nut Vulture	LC	2.00	0.99	391	782.00
	Group Totals					4,123	5,702.30
Carnivora							
	*Nandinia binotata* ^a^	African Palm Civet	LC	2.95	0.00	1,241	3,660.95
	*Poiana richardsonii* ^a^	African Linsang	LC	0.60	0.11	588	352.80
	Group Totals					1,829	4,013.75
Hyracoidea							
	*Dendrohyrax dorsalis* ^d^	Western Tree Hyrax	LC	3.00	1.00	1,553	4,659.00
Pholidota							
	*Manis tricuspis* ^a^	Tree Pangolin	NT	1.50	0.50	6,302	9,453.00
	*Smutsia gigantea* ^b^	Giant Ground Pangolin	VU	32.50	0.00	118	3,835.00
	Group Totals					6,420	13,288.00
Primates							
	*Allochrocebus preussi insularis* * ^e^	Bioko Preuss's Monkey	EN	4.50	1.00	2,142	9,639.00
	*Cercopithecus erythrotis erythrotis* * ^e^	Bioko Red-eared Monkey	VU	3.40	1.00	17,997	61,189.80
	*Cercopithecus nictitans martini* ^e^	Stampfli's Putty-Nosed Monkey	LC	4.60	0.98	336	1,545.60
	*Cercopithecus pogonias pogonias* ^e^	Golden-bellied Crowned Monkey	LC	3.25	1.00	2,720	8,840.00
	*Colobus satanas satanas* * ^e^	Bioko Black Colobus	VU	9.25	1.00	5,122	47,378.50
	*Mandrillus leucophaeus poensis* * ^e^	Bioko Drill	EN	14.25	1.00	5,004	71,307.00
	*Procolobus pennantii* ** ^f^	Pennant's Red Colobus	CR	10.50	1.00	1,754	18,417.00
	*Sciurocheirus alleni alleni* * ^a^	Bioko Allen's Galago	LC	0.26	1.00	160	41.60
	Group Totals					35,235	218,358.50
Reptilia							
	*Kinyxis erosa* ^b^	Serrated Hinge-back Tortoise	DD	1.00	0.00	3,323	3,323.00
	*Osteolaemus tetraspis* ^b^	Dwarf Crocodile	VU	7.50	0.00	1,913	14,347.50
	*Python sebae* ^c^	African Rock Python	DD	13.83	1.00	1,001	13,841.41
	*Varanus niloticus* ^b^	Nile Monitor	DD	6.50	0.45	1,618	10,517.00
	Group Totals					7,855	42,028.91
Rodentia							
	*Atherurus africanus* ^a^	African Brush-tailed Porcupine	LC	2.83	0.91	24,302	68,798.96
	*Cricetomys emini* ^a^	Emin's Pouched Rat	LC	1.14	1.00	44,624	50,871.36
	*Myosciurus pumilio* ^d^	African Pygmy Squirrel	LC	0.02	1.00	105	2.10
	*Protoxerus stangeri* ^d^	African Giant Squirrel	LC	0.77	1.00	9,967	7,674.59
	*Thryonomys swinderianus* ^d^	Greater Cane Rat	LC	6.65	0.00	1,914	12,728.10
	Group Totals					80,912	140,075.11
Totals						196,892	873,993.28

Species were omitted from all analyses due to rarity if enumerated less than 75 times, and are not included in the table. Biomass values taken from:

^a^ Fa and Purvis [75],

^b^ Juste et al. [76],

^c^ Willcox and Nambu [77],

^d^ Kingdon [78],

^e^ Butynski et al. [79],

^f^ Groves [80]. Values from Butynski et al. [79] are an average of the reported weights for both sexes.

^†^ Adapted from the IUCN Red List of Threatened Species [81]: CR critically endangered, EN endangered, NT near threatened, VU vulnerable, LC least concern, DD data deficient.

^‡^ Proportion of carcasses from Bioko compared to Mainland.

* Recognized by Grubb et al. [82] as subspecies endemic to Bioko.

** Recognized by Groves [80] as species endemic to Bioko.

Rates of trapped and shotgunned carcasses were negatively correlated [r = -0.86 (95% CI -0.9079, -0.8198)], specifically in the Pre-ban and Post-ban periods as the market grew and became more profit-oriented (Fig 2a, S1 Fig). Furthermore, in a two-factor ANOVA, the interaction of carcass rates with capture method and period was highly significant (F_2,306_ = 227.23, MSE = 0.109, p < 0.001), as mean carcass rates increased for both trap and shotgun over the Early and Pre-ban periods, but only increased for shotgun Post-ban (S1 Fig).

Domestic carcasses make up the majority of the market volume, but as early as September 2003, imported carcasses began occurring regularly at the market (Fig 2b). Rates of imported carcasses increased through September 2010, peaking just before the end of the study in June 2010. Although the primate hunting ban was not intended to affect other taxa, the domestic carcass rate declined rapidly following its publication (Fig 2).

The mean carcass rate of primates increased across all three periods from 4.26 carcasses/day (SD 1.82) to 9.51 carcasses/day (SD 3.32) and ultimately to 17.11 carcasses/day (SD 11.25). The primate carcass rate increased steadily throughout Pre-ban, but decreased to nearly zero (0.77 carcasses/day) following the publication of the primate hunting ban in November 2007. Shortly after the decline, the primate carcass rate increased rapidly (Fig 2c) reaching a maximum of 37.42 carcasses/day in April 2010. From July through September 2010, the primate carcass rate declined by over 30% to 24.26 carcasses/day.

### Game harvest profile

By taxonomic groups, 88.94% of carcasses were either rodents (n = 80,912, 41.09%), ungulates (n = 58,965, 29.95%), or primates (n = 35,235, 17.90%). The two duiker species, including blue duiker (*Philantomba monticola*) and Ogilby’s duiker (*Cephalophus ogilbyi*), represented 29.81% (n = 58,699) of the overall market. Reptiles (n = 7,855, 3.99%), pangolins (n = 6,420, 3.26%), and birds (n = 4,123, 2.09%) represented the other important groups in the market (Table 2). The most abundant species was *P*. *monticola*, representing 25.05% of all carcasses, followed by Emin’s pouched rat (*Cricetomys emini*, 22.66%), the brush-tailed porcupine (*Atherurus africanus*, 12.34%), and the Bioko red-eared guenon (*Cercopithecus erythrotis erythrotis*, 9.14%). Both *P*. *monticola* (27.65%) and *C*. *ogilbyi* (20.93%) contributed significantly greater proportions of the overall market biomass, more than doubling the third highest amount contributed by the Bioko drill, (*Mandrillus leucophaeus poensis*) (8.16%) (Table 2). Overall, 92.03% of the market biomass was comprised of ungulates (51.02%), primates (24.98%), and rodents (16.03%), with the two duiker species making up almost half of the total market (48.58%). The remaining market biomass consisted of reptiles (4.81%), pangolins (1.52%), birds (0.65%), hyraxes (*Dendrohyrax dorsalis*) (0.53%), and carnivores (0.46%).

### Intervention Model

**Fig 3:**
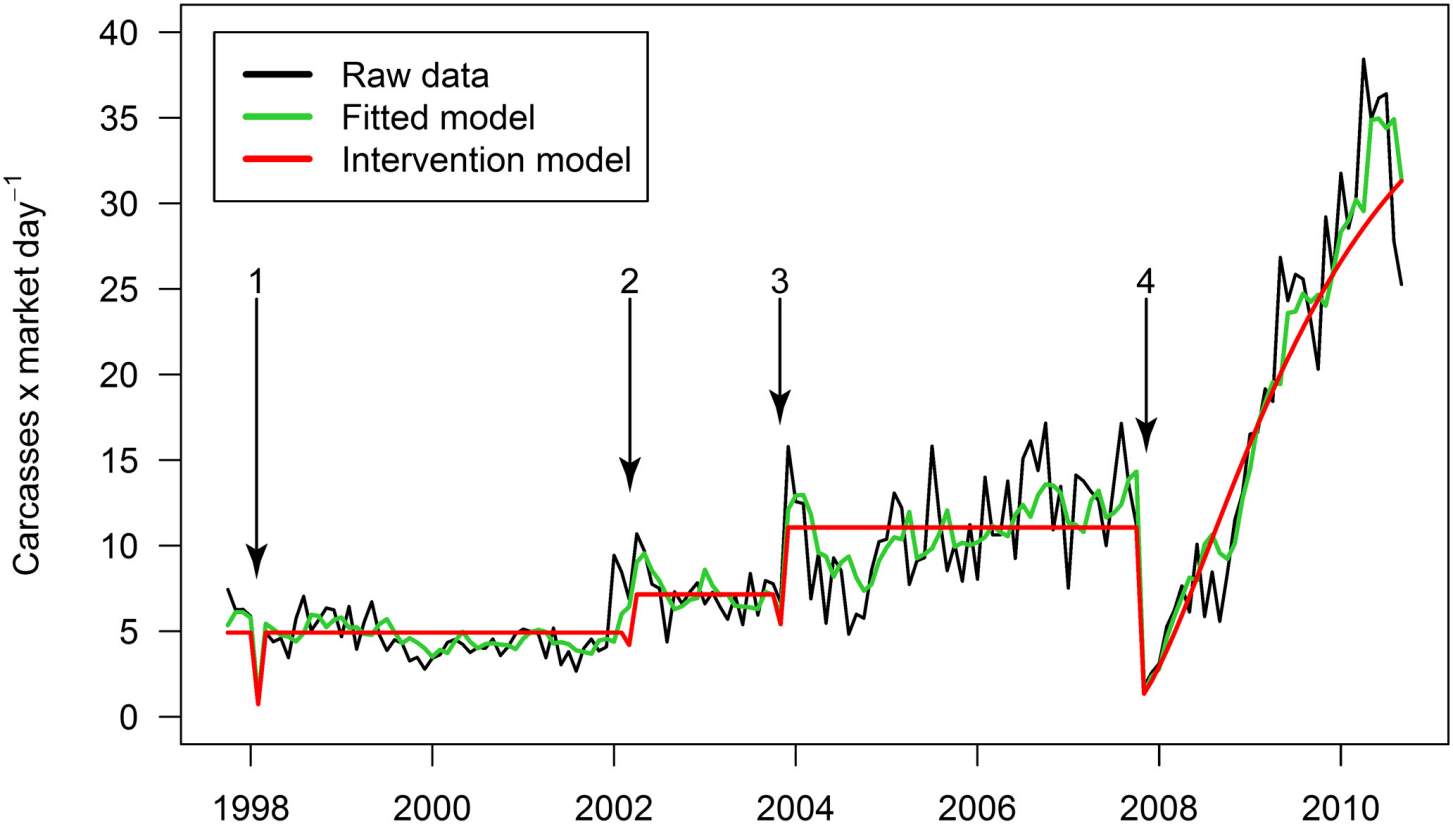
Primate carcass rates raw data, fitted, and predicted values. Values are as estimated in the intervention analysis via ARIMA (2,0,0,)x(1,0,0)_12_. Significant interventions are numbered at: 1) 2/1998 –political uprising; 2) 3/2002 –bushmeat roundtable; 3) 11/2003 –Law 7/2003; 4) 11/2007 –Decree 72/2007.

The pre-intervention SARIMA model consisted of a second order autoregressive term (*ϕ* = 0.343 (*SE* = 0.078) and 0.301 (0.078)) and a first order seasonal autoregressive term with period 12 (0.208 (0.084)): *ARIMA*(2, 0, 0)*x*(1, 0, 0)_12_. The model indicated that four out of the five notable events during the study period significantly altered market dynamics (RMSE 0.323; AIC 115.03) (Table 1; Fig 3). Intervention 2/1998 consisted of only a downward pulse, a momentary deviation that rapidly returned to baseline; whereas, 3/2002 and 11/2003 began as a downward pulse, but were followed by a step, a permanent shift in the mean of the series. The primate hunting ban was the most dramatic market intervention, decreasing the primate carcass rate by 87.81% (95% CI: 79.75, 95.88) between October and November 2007 (Fig 3) via an initial pulse. Its time lag parameter, beginning at a local minimum caused by the pulse function, determines the number of time steps required to reach the significantly increased mean carcass rate at the end of the series, determined by the step function; that is, the lag accounts for the gradual increase in primate carcass rates from11/2007 through 9/2010 (Table 1; Fig 3).

### Trend analysis

**Fig 4:**
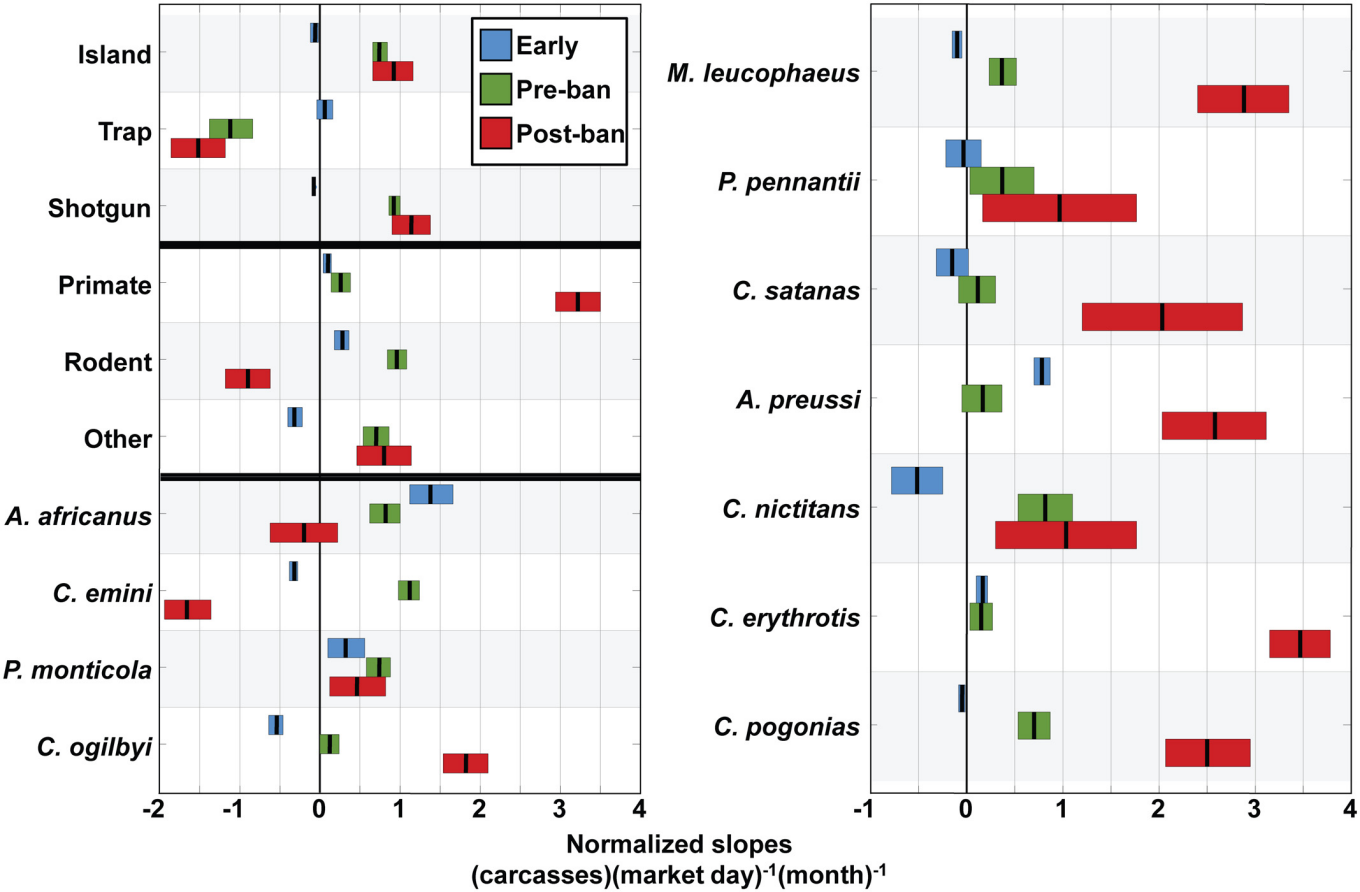
Average change in carcass rate per month (slopes) and 95% confidence intervals for each aggregation, taxon, and period. Included for analysis were all species originating on Bioko that comprised >0.01% of the total market biomass (arbitrary threshold). Slopes were normalized by the ratio of standard deviations for the entire time series of the respective taxa and total carcasses in order to directly compare the fractional representation of the taxon in the total market carcass rate. No imported species were included for analysis.

No significant trend was observed in the Early period in total number of carcasses, independent of species or taxonomic group (-0.043 carc md^-1^ mo^-1^, CI 0.040) (Fig 4). This lack of trend was present in all aggregate groups. All Pre-ban trends in aggregate groups were significantly different from their Early counterparts. Of these aggregate groups, only trapped carcasses exhibited a decreasing Pre-ban trend (-1.102 carc md^-1^ mo^-1^, CI 0.265) (Fig 4)

Overall, there was a significant change in the proportional representation of taxonomic groups in the market in the Post-ban period (Figs 2 and 4). Primates increased at a slope nearly 2.6 times greater than other aggregate taxa (3.227 carc md^-1^ mo^-1^, CI 0.285), indicative of the rapid post-ban increase in primate carcasses (Fig 4). The rate of rodent carcasses decreased in Post-ban (-0.895 carc md^-1^ mo^-1^, CI 0.279) and was significantly lower than in Early (0.281 carc md^-1^ mo^-1^, CI 0.082) and Pre-ban (0.968 carc md^-1^ mo^-1^, CI 0.114). This is largely related to the observed decrease in trap rates since 59.3% of rodents were captured via trapping. Avian carcasses also decreased from Pre-ban (0.743 carc md^-1^ mo^-1^, CI 0.119) to Post-ban (-0.493 carc md^-1^ mo^-1^, CI 0.464). For the total market, however, the increasing trend in the total number of carcasses during Pre-ban (0.766 carc md^-1^ mo^-1^, CI 0.089) did not significantly change Post-ban (0.782 carc md^-1^ mo^-1^, CI 0.207).

Interspecific analysis provided additional insight into the large rate changes in the market in the Post-ban period. Of the seven primate species analyzed (Bioko Allen’s galago [*Sciurocheirus alleni*] was omitted due to small sample size), five showed significantly greater slopes Post-ban compared to the other periods (Fig 4). For instance, in Post-ban, *C*. *erythrotis*, which comprised 51.1% of all primates, had the steepest slope in the analysis (3.475 carc md^-1^ mo^-1^, CI 0.317), essentially driving the large Post-ban increase in the primate group. In contrast, *C*. *emini*, *A*. *africanus*, Nile monitor (*Varanus niloticus*), and *M*. *tricuspis* were all predominantly trapped (70.2%, 54.0%, 69.6%, 89.9% of carcasses, respectively), and contributed to the rapid decrease in Post-ban trapping rates (Fig 4).

### Correlation between consumption and increased incomes

Rates of shotgunned carcasses were positively correlated with oil price in the Pre-ban and Post-ban periods (Table 3), supporting the hypothesis that bushmeat may have emerged as a ‘luxury’ good on Bioko as wealth and disposable income increased [29].

**Table 3:** Regression of average carcass rates for total, shotgunned, and trapped carcasses with oil prices as a proxy for income in Early, Pre-ban, and Post-ban periods.

Taxa	Period	Intercept	Slope	*R* ^*2*^	*P*
Total	Early	35.40	-0.249	0.151	0.07
	Pre-ban	15.96	0.619	0.597	0.00*
	Post-ban	96.83	-0.233	0.256	0.09
	All	19.76	0.605	0.610	0.00*
Shotgun	Early	13.19	-0.141	0.107	0.14
	Pre-ban	-18.61	0.820	0.605	0.00*
	Post-ban	91.35	-0.363	0.332	0.05
	All	-2.39	0.643	0.536	0.00*
Trap	Early	21.81	-0.171	0.161	0.06
	Pre-ban	33.71	-0.188	0.117	0.16
	Post-ban	5.33	0.131	0.448	0.02*
	All	20.25	-0.014	0.003	0.69

* indicates significance level *p* < 0.05.

## Discussion

Bushmeat market surveys are an important and cost-effective tool for assessing the extent and dynamics of the commercial bushmeat trade [14, 39, 83]. Our work represents one of the longest running, continuous studies of bushmeat market dynamics in the west and central African forest zone, encompassing temporal fluctuations in climate (multiple wet-dry seasons), which can affect hunting patterns [66, 84, 85], economic development (Equatorial Guinea’s emergence as a major hydrocarbon exporter), changes in policy (i.e., bans on primate hunting and consumption), and major socio-political events (failed attempted coups in 1998, 2004 and 2009). The value of this dataset stems from the fact that few studies are conducted continuously over long enough periods of time to be able to assess long term trends in market dynamics (but see Crookes et al. [57]). Previous studies have used a ‘snapshot’ [41, 76] approach to support (or refute) the efficacy of various conservation measures. These types of studies are important to describe the general characteristics of markets (e.g. Fa et al. [38]), and, if replicated, to provide information on general patterns over time (e.g. Fa et al. [41], Albrectsen et al. [39], and Allenbone-Webb et al. [83]), data which are often critical to informing conservation measures. In contrast, however, our long-term data set combined with appropriately applied time series analyses, allowed us to identify subtle changes in the market over time that we would otherwise have been unable to detect, such as the short- and long-term effects of market interventions, species-specific hunting patterns within taxonomic groupings, and seasonality in hunting patterns across several years [66]. There are, of course, many limitations to the application of market data, arising primarily due to confounding factors [57], but by restricting our in-depth analyses to only Bioko taxa, we were able to control for catchment size, and although bushmeat is consumed in villages throughout Bioko [44, 66], the majority of hunted game on the island is traded through the Malabo market [39, 86]. Data collected from long-term studies such as this are important for inferring temporal changes in hunting intensity, bushmeat demand, and the sustainability of wildlife hunting, and can be used to gauge the impact of conservation initiatives [41, 57, 87].

Our results indicate consistent growth in the bushmeat market that was concurrent with the dramatic socioeconomic transformation of Equatorial Guinea. With rising incomes [29, 39], demand for fresh meat increases [88], leading to higher prices and greater potential profit for bushmeat carcasses. As a result, the market for profitable bushmeat hunting grew, which was accompanied by a transition from trapping game to shotgun hunting, a pattern also observed in Rio Muni [89]. Gun hunting allows for greater prey choice, provides a higher return on invested time, and permits the market supply chain better coordination of hunting and transport, relative to trapping, a more passive form of hunting [90]. Hunters can organize efforts to coincide with transport, which when combined with greater selectivity, maximizes their potential to deliver fresh carcasses to market. Shotgun hunting has also become more affordable over time [91], allowing hunters to target smaller prey (e.g., rodents) without fear of a net loss. For example, adjusting for inflation, one pouched rat (*Cricetomys emini*), with an average 2010 price of 3,940 FCFA (approximately 7.90 USD), can earn a hunter approximately 27 times the expected profit in 1997. Mainland carcasses also began appearing regularly in the market in the Pre-ban period (Fig 2b), suggesting that the costs and risks associated with carcass transport are largely negated by higher profit potential in the Malabo market relative to markets in Nigeria, Cameroon, or Rio Muni [29]. Mainland carcasses (e.g., hinge-back tortoises [*Kinyxis erosa*], greater cane rats [*Thryonomys swinderianus*]) are now commonly shipped to Malabo for sale, aided by routine air and ship travel between Bioko and the mainland.

The rise in gun hunting has been concurrent with higher carcass rates of Bioko’s threatened monkeys, the taxa which best illustrate the negative effects government intervention has had on inhibiting the bushmeat trade. Following the 2002 Biodiversity Roundtable, primate carcass rates more than doubled, beginning a steady increase that ended with the enactment of the primate ban in October 2007. The Post-ban period began with the immediate crash in primate carcass rates (Fig 2c) because vendors and consumers alike initially complied with the Presidential decree. However, lacking enforcement of the ban, compliance soon gave way to hunting that exceeded Pre-ban levels (Fig 2c). This pattern suggests a “mardi gras” mentality, in which market players sought to exploit a given resource before the potential effects of legislation could take hold. In doing so, suppliers could maximize short-term profits by increasing supply to meet high demand from consumers who recognize the resource may soon be unavailable. Although not previously shown for the bushmeat trade, a similar pattern has been described in species uplisted from CITES Appendix II to Appendix I, in which trade volumes peaked in the transition period between submission of the uplisting proposal and when it took effect [32].

Our data also support the hypothesis that higher incomes resulting from the petroleum boom [29, 39, 43], and the emergence of a relatively ‘luxury’ market driven by rising immigration of people non-native to Bioko with a cultural preference for bushmeat [29, 88, 92] have contributed to the continuously increasing demand for bushmeat we observed in the market. This increasing demand coupled with the growth of the urban population in Malabo have driven up the commercial value of market animals, increased bushmeat profitability [29], and led to increased commercialization of the overall market. Consumers are willing to pay a premium for bushmeat over other sources of animal protein [29, 43, 88], which normally would preclude non-luxury consumers from purchasing those goods. However, elsewhere in central Africa even middle class consumers are participating in the market [93].

Market growth has varied among taxa. For instance, although the total carcass rate increased from Pre-ban to Post-ban, the rate of change in carcass rate per month (slopes) of many taxa decreased. Capture of taxa typically taken by shotgun, primates and the larger Ogilby’s duiker (*Cephalophus ogilbyi*) (99% and 79% shotgunned, respectively) surged, while slopes of typically trapped taxa declined (Fig 3). Not all primate taxa mirrored the aggregate trend. Pennant’s red colobus (*Procolobus pennantii*) and the Bioko putty-nosed guenon (*Cercopithecus nictitans martini*), both of which have population densities highest in the remote southwest sector of Bioko, showed no significant change in slope from Pre-ban to Post-ban (Fig 4). This suggests that the potential profits to be gained from these species may be less than the costs associated with bringing them to the market, and that isolation and the long-term research presence of BBPP and UNGE in the GCSR may have contributed to their protection from significant exploitation, despite an intensification of primate hunting elsewhere. Furthermore, given the isolated extent of their ranges, these species can be used an indicator of hunting activity in remote areas of the GCSR. For example, the range of *P*. *pennantii* is restricted to the remote southwest of the GCSR [37, 40, 49, 94, 95], and where it does occur, *P*. *pennantii* are easy to detect [37, 49, 96, 97]. As such, if we do not observe any individuals in the market, we can assume that hunting is either absent or occurring at only minimal levels in the area.

Whatever protection is afforded via isolation is quickly being stripped away by infrastructure development. A recently completed road now bisects the Gran Caldera Scientific Reserve, connecting Malabo with the largely unaffected southern beaches in under two hours. The road gives bushmeat hunters and turtle poachers rapid direct overland access to the remote forests of the GCSR and the beaches of the southern coast, which were previously only accessible on foot or by boat. Unfortunately, “avoiding the first cut” [98] within the GCSR is no longer possible, and in addition to the inevitable influx of colonization and development [99], we predict an increase in the numbers of primates and other bushmeat taxa, as well as marine turtles, originating in the remote southern sector of that GCSR that end up in the Malabo market and restaurants.

Securing the long-term future of Bioko’s biodiversity will require (*i*) development and implementation of adaptive, evidence-based management plans for Bioko’s protected areas; (*ii*) strengthening of the legal basis for the protected areas; (*iii*) empowerment of the National Institute of Forestry Development and Protected Area Management (INDEFOR-AP) and Ministry of Fisheries and the Environment, the federal entities tasked with management of protected areas, via increased budgets, institutional support, and authority to enforce environmental legislation; (*iv*) increased law enforcement effectiveness, including increased presence and activities in protected areas, regular monitoring of protected areas, transit routes, and markets, and arrests and prosecutions of poachers; and (*v*) committed involvement from the Government of Equatorial Guinea in order to not only stop illegal hunting, but also to mitigate impacts from its ambitious and unregulated infrastructure development plans. More immediate short-term measures, such as enforcement of existing legislation, could be taken by the Government of Equatorial Guinea, putting into effect barriers to the bushmeat trade which could significantly reduce the amount of primate hunting. The primate hunting ban, for instance, includes prohibitive fines (100,000–500,000 FCFA/monkey) [50] which, if enforced, would deter hunters by threatening a significant portion of their annual hunting income (~240,000 to 934,000 FCFA/year) [43, 44]. Enforcement could begin at preexisting roadblocks on the two direct routes between catchment areas and Malabo, and could begin to control the transport of primate carcasses via confiscation and fines [100, 101]. Perhaps the most practical expeditious solution, however, would be the implementation of forest guards [6], a successful strategy, that has been linked to reductions in hunting and improved effectiveness of protected areas [102–104]. Long-term management planning is critical, but rapid, effective, and immediate solutions, such as forest guards, are urgently needed to safeguard Bioko’s protected areas, and to stop the extensive hunting of threatened taxa, such as *C*. *ogilbyi* and diurnal primates, that together contribute over 50% to the overall market biomass (Table 2), yet are already declining in numbers around villages and on the peripheries of supposed protected areas [66].

In addition to enforcement strategies to diminish supply, alternative measures have also been presented to manage and potentially reduce demand for bushmeat. Grande Vega et al. [44], for example, have presented the case for the development of a wildlife farming industry on Bioko focused on large rodents and *P*. *monticola*, provided that it was well-regulated. Our results suggest that if considering demand alone, there may be merit to this suggestion. It could potentially help to alleviate urban demand, since high volumes of species preferred by consumers (*C*. *emini*, *A*. *africanus*, and *P*. *monticola)* were observed, all of which have high intrinsic growth rates [92]. However, the feasibility of captive rearing has been questioned while wild individuals exist as essentially a free good [105, 106], despite the necessity of forward-thinking solutions that adapt to local drivers of demand. Furthermore, the introduction of any sort of wildlife farming on Bioko would rely heavily on effective regulatory enforcement, of which there has been little to none on Bioko. This begs the question of whether farming wildlife on Bioko is practical, when short-term goals, such as forest guards, have been conspicuously ignored for years despite numerous calls for their implementation [37, 39, 40, 42, 43, 107].

Ultimately, for any enforcement strategy to be successful, it must have support from the government [6, 108]. Our study documents that in spite of some governmental conservation planning and legislation, the bushmeat market on Bioko has continued to grow over time, becoming more commercial and increasingly detrimental to Bioko’s primates [66]. The data also suggest a path forward to reduce the supply of bushmeat: legislation by the government of Equatorial Guinea can strongly affect the dynamics of the bushmeat market. Following each intervention, there was a dramatic decrease in carcass availability in the market (Fig 4). For example, following the decree banning primate hunting, the number of primate carcasses in the market dropped to nearly zero. This effect, however, is only short-term, since in the absence of obvious enforcement of the decree, the market quickly rebounded in 2008, exceeding previous levels. These observations suggest that further legislative action by the government of Equatorial Guinea may aid in reducing hunting pressure, but also highlight the importance of follow through and improved law enforcement by government agencies in order to effectively conserve wildlife. With sufficient political will, as well as an expansion of collaborative conservation efforts via institutions like UNGE engaging with communities, it may yet be possible to conserve the wildlife and habitats of Bioko, especially in the GCSR. Within the GCSR boundaries, relatively high primate densities persist [66] despite rapid recent infrastructure development lacking environmental impact assessments and oversight by the Equatoguinean government’s environmental agencies.

There is no panacea for addressing bushmeat hunting, nor is there a conservation paradigm robust enough to effectively account for all of the complex and interconnected socio-political factors that drive wildlife exploitation. However, the trends presented here cannot persist indefinitely. Under pressure from human population growth and increasing development, demand for and access to bushmeat will continue to rise, and populations of many game species on Bioko, especially primates, will likely collapse in the face of increasing offtake levels. All stakeholders on Bioko must work together to develop and implement effective and enduring solutions in the immediate future, in order to preserve the rich biodiversity of Bioko.

## Supporting Information

S1 Fig Interaction plots of a two-factor analysis of variance (ANOVA) showing the effect of capture method (i.e., shotgun or trap) and period on carcass rates. We performed a two-factor analysis of variance (ANOVA) to determine how capture method and period affect rates. Capture rates have been log transformed to reduce residual heteroscedasticity.
(TIF)
