# Correction: Long-Term Urban Market Dynamics Reveal Increased Bushmeat Carcass Volume despite Economic Growth and Proactive Environmental Legislation on Bioko Island, Equatorial Guinea

**DOI:** 10.1371/journal.pone.0137470

**Published:** 2015-08-28

**Authors:** The PLOS ONE Staff

## Notice of Republication

This article was republished on August 19, 2015, because part of Table 2 was erroneously omitted during the typesetting process. The publisher apologizes for the errors. Please visit this article again to view the correct version. The PDF of this article was not affected.
