# Supplementary figures and images for: Long-Term Urban Market Dynamics Reveal Increased Bushmeat Carcass Volume despite Economic Growth and Proactive Environmental Legislation on Bioko Island, Equatorial Guinea
Source: PLoS One. 2015 Jul 31;10(7):e0134464. doi: 10.1371/journal.pone.0134464 (PMC4521855; doi:10.1371/journal.pone.0134464)

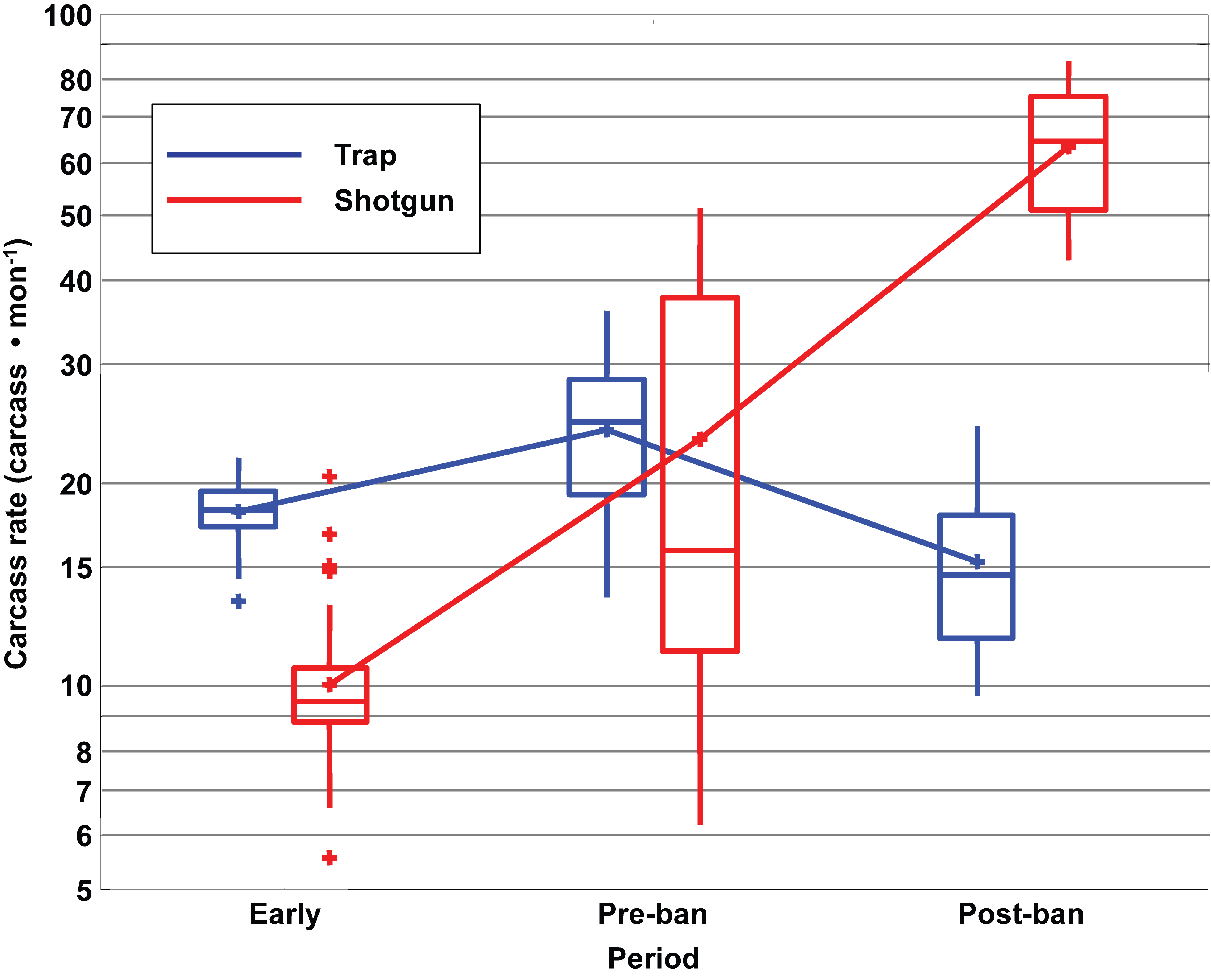

Supplement: S1 Fig — We performed a two-factor analysis of variance (ANOVA) to determine how capture method and period affect rates. Capture rates have been log transformed to reduce residual heteroscedasticity. (TIF) [file pone.0134464.s001.tif]
